# Associations between thyroid function and gestational diabetes mellitus in Chinese pregnant women: a retrospective cohort study

**DOI:** 10.1186/s12902-022-00959-y

**Published:** 2022-02-21

**Authors:** Geng-dong Chen, Xiao-Yan Gou, Ting-ting Pang, Peng-sheng Li, Zi-xing Zhou, Dong-xin Lin, Da-zhi Fan, Xiao-ling Guo, Li-juan Wang, Zheng-ping Liu

**Affiliations:** 1 Foshan Institute of Fetal Medicine, Affiliated Foshan Maternity & Child Healthcare Hospital, Southern Medical University, No.11 Renmin West Road, Foshan, 528000 Guangdong China; 2 The Second School of Clinical Medicine, Southern Medical University, Guangzhou, 510515 Guangdong China; 3 Department of Medical Records, Child Healthcare Hospital, Southern Medical University, Affiliated Foshan Maternity &, Foshan, 528000 Guangdong China; 4 Department of Obstetrics, Changchen District, Child Healthcare Hospital, Southern Medical University, Affiliated Foshan Maternity &No.11 Renmin West Road, Foshan, 528000 Guangdong China

**Keywords:** Free tetraiodothyronine, Thyroid-stimulating hormone, Thyroid peroxidase antibody, Thyroid function, Gestational diabetes mellitus

## Abstract

**Background:**

Thyroid function is known to be closely linked with type 2 diabetes, but data on the association between thyroid function and gestational diabetes mellitus (GDM) are inconsistent.

**Methods:**

A total of 2849 pregnant women were included in this retrospective study. Serum concentrations of thyroid indicators (free tetraiodothyronine, FT4; thyroid-stimulating hormone, TSH; and thyroid peroxidase antibody, TPO Ab) were obtained from a clinical laboratory. The presence of GDM were drawn from medical records. The clinical subtypes of thyroid function (euthyroidism, subclinical hypothyroidism, hyperthyroidism, and isolated hypothyroxinemia) were categorized according to the thresholds of the 2.5^th^/97.5^th^ and 10^th^/90^th^ percentiles of TSH and FT4 concentrations. A concentration of > 34 IU/L was defined as indicating TPO Ab-positivity.

**Results:**

Two hundred and thirty-five (8.25%) of the 2849 women were TPO Ab-positive. Higher serum concentrations of FT4 (top vs. bottom tertiles) was found to be negatively associated with the risk of GDM. The corresponding *odds* (*OR)* values (top tertile vs. bottom tertile) were 0.71 [*95% confidence interval (CI)*: 0.54, 0.93]. No significant associations were observed between the extremely 2.5^th^/97.5^th^ or 10^th^/90^th^ percentiles of FT4 concentration, TSH concentration, thyroid function subtypes (vs. euthyroidism), TPO Ab-positivity (vs. -negativity), and the GDM risk. The corresponding results remained similar when TPO Ab-positive subjects were excluded.

**Conclusions:**

A negative association with the risk of GDM was observed for the highest FT4 concentrations tertile. No significant associations were found between the TSH concentration, thyroid function subtypes, TPO Ab positivity, and the GDM risk.

**Supplementary Information:**

The online version contains supplementary material available at 10.1186/s12902-022-00959-y.

## Background

Gestational diabetes mellitus (GDM) is a major complication that may occur during pregnancy and greatly affects the health of mothers and infants [1]. Mothers with GDM have a higher risk of adverse birth outcomes [2, 3], and may be imposed to a seven-fold increased risk of type 2 diabetes mellitus (T2DM) in later life [4]. The prevalence of GDM has progressively increased in China, and now occurs at a rate of 14.8% according to a recent report [5]. More studies are urgently needed to better understand the potential pathogenesis and risk factors for GDM and thus identify possible control measures.

Thyroid hormones are important for maintaining the current and long-term health of all humans, including that of mothers [6] and children [7–9]. Several indicators are commonly used to measure thyroid function, namely the concentrations of free thyroxine (FT4), thyroid-stimulating hormone (TSH), and anti-thyroperoxidase antibody (TPO Ab). A person’s thyroid status (e.g., euthyroidism; subclinical hypothyroidism, SCH; isolated hypothyroxinemia; or hyperthyroidism) can be determined according to their relative concentrations of FT4 and TSH [10].

A certain proportion of women suffer thyroid disorders during pregnancy. For instance, the reported prevalence rates of TPO Ab positivity and SCH were 7.5% and 3.1%, respectively, in a recent meta-analysis study [9]. Thyroid disorders are closely related with diabetes disorder; as the prevalence of thyroid disorders increases in women with type 1 or type 2 diabetes mellitus, and thyroid disorders also increase the risk of T2DM [11]. However, although several studies have been performed, no consistent conclusions have been drawn to illustrate the influence of thyroid disorders on the occurrence of GDM [6, 12–21].

Higher FT4 concentrations were found to protect against GDM in a large retrospective study of 27,513 Chinese women [20]; however, two studies in the US did not identify any significant associations [16, 21]. Similarly, other studies have reported positive [13, 22], negative [20], or no associations [6, 16, 17] between the TSH concentration and GDM. In a large retrospective study, TSH concentrations (≤ 3.2 mIU/L) were adversely related with GDM, but such relationship disappeared in those with a TSH concentration > 3.2 mIU/L [13]. The use of different TSH concentration cutoff points to define TSH abnormality in previous studies might increase the difficulties in comparing these results. In order to attenuate the potential influence of different race or population, the extremely 2.5^th^/97.5^th^ percentile of FT4 or TSH were also classified as abnormality [8, 23]. These former evidences might encourage the use of multiple classified ways of thyroid indicators in a same study to increase the comparability.

Both hypothyroidism/SCH [12, 24, 25] and TPO Ab-positivity [14, 19, 26] were suggested to be associated with a higher risk of GDM, and this risk is further increased when these statuses co-occur [24, 27]. However, this pattern could not be supported in several other studies, as no significant associations between these factors were identified [14, 17, 18, 20]. Only one or two thyroid disorder status (mostly subclinical hypothyroidism) were investigated, while other thyroid disorders were neglected in these studies [12, 14, 22, 24]. More studies based on full-scale thyroid indicators and disorders are required to enrich the literature and better explore this field.

Therefore, in this retrospective study, we aimed to investigate the associations of thyroid function indicators (FT4, TSH, and TPO Ab) and thyroid disorders with the occurrence of GDM in Chinese pregnant women.

## Material and methods

### Subjects

**Fig. 1:**
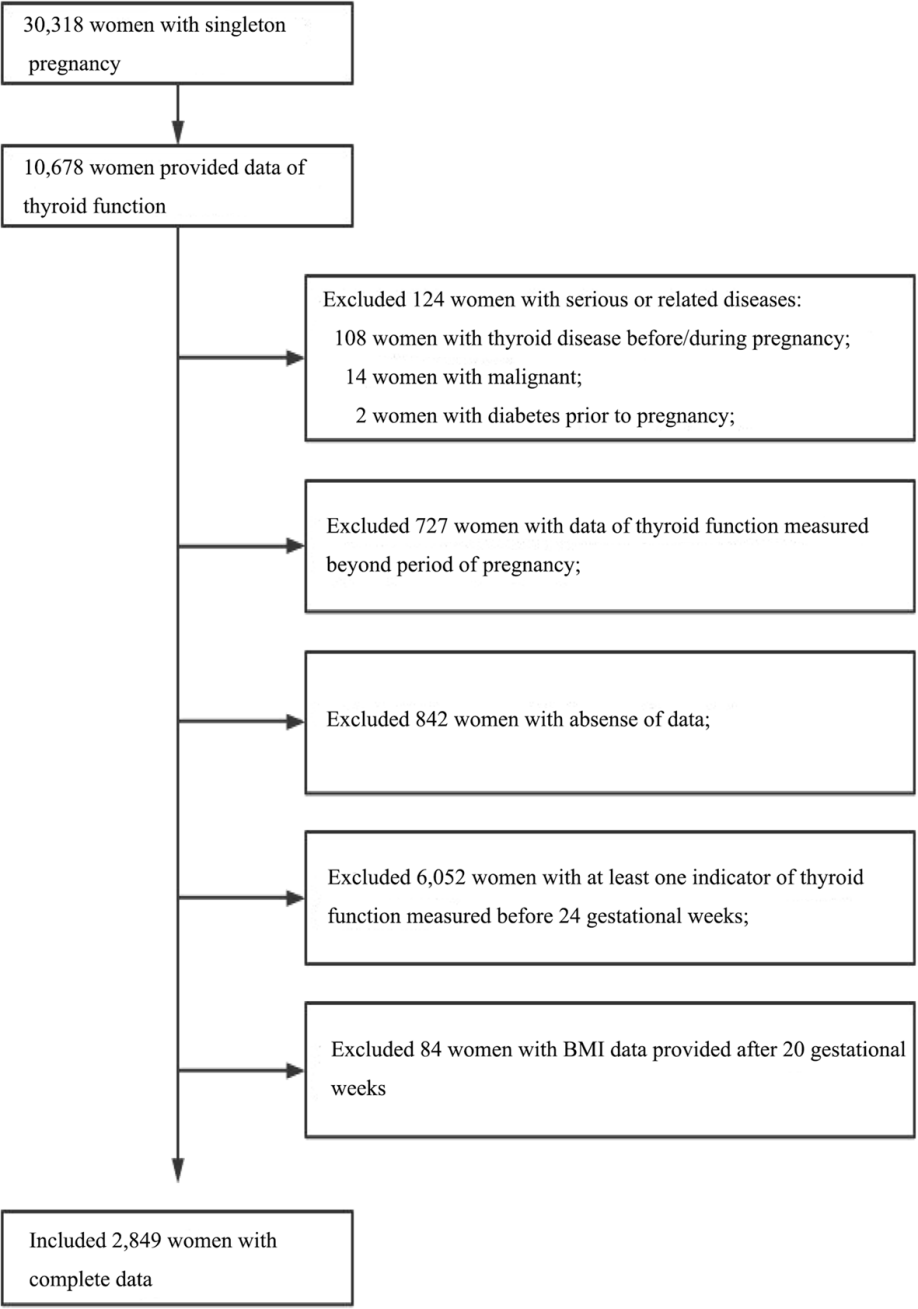
Flow chart of study participants

This study was performed at a large obstetric center in Foshan City, Guangdong Province, China (Affiliated Foshan Maternity & Child Healthcare Hospital, Southern Medical University). Medical data from March 1, 2015 to July 31, 2018 were retrospectively reviewed. Subjects aged 18 to 45 years whose records contained data on the exposure (thyroid function: FT4, TSH, and TPO Ab concentrations) and outcome (GDM) were included in the study. The details of flow of participants were shown in Fig. 1. To be brief, 10,678 out of 30,318 singleton pregnant women provided data of thyroid function. Subjects with the following situation were excluded: (1) history of diagnosed thyroid diseases and other serious disease (124 women), including all types of cancer, T2DM, and cardiovascular disease; (2) data of thyroid function measured beyond period of pregnancy (727 women); (3) the lack of any exposure indicators or outcome data (842 women); (4) collection of thyroid function measurements subsequent to an oral glucose-tolerance test (OGTT) before 24 gestational weeks (6,052 women); and (5) BMI information provided (at first obstetric check-up) after 20 gestational weeks (84 women). Finally, a total of 2849 pregnant women with all the required data were included in this retrospective study.

### Thyroid function

Three indicators that reflect thyroid function (FT4, TSH, and TPO Ab concentrations) and were measured during the regular obstetric check-ups in the same clinical laboratory were reviewed in this study. Blood samples were collected by nurses and immediately measured in the same laboratory of the hospital without being frozen. Serum concentrations of FT4, TSH, and TPO Ab were measured using an electrochemical luminescence automatic immune analyzer (Cobas e601, Roche Inc, Switzerland). The gestational weeks at the time of measurement of these indicators was also reviewed, and thyroid function measurements that had been taken subsequent to an OGTT were excluded to avoid causal inversion associations. Measurements were divided into tertile groups (with the bottom tertile group representing the lowest values and the top tertile group representing the highest values), extremely 10^th^ percentile groups (< 10^th^, 10 ~ 90^th^, > 90^th^ percentile) according to their values in our population. Subjects were further divided in to extremely 2.5^th^ percentile (< 2.5^th^, 2.5 ~ 97.5^th^, > 97.5^th^ percentile) according to the reference in Chinese pregnant women in a local guideline [23].

To further investigate the associations between different thyroid statuses and to enable a better comparison of the data with those of other studies, subjects were further divided into different groups (euthyroidism, subclinical hypothyroidism, or hyperthyroidism) based on the extremely 2.5^th^/97.5^th^ percentiles of TSH and FT4 reference from a local guideline [23], according to the criteria published before [8]. Briefly, (1) euthyroidism was defined as a TSH and FT4 concentration within the normal range of 2.5^th^ to 97.5^th^ percentile; (2) subclinical hypothyroidism was defined as a TSH concentration > 97.5^th^ percentile and a FT4 concentration within the normal range; (3) overt hyperthyroidism was defined as a TSH concentration < 2.5^th^ percentile and an FT4 concentration > 97.5^th^ percentile, while subclinical hyperthyroidism was defined as a TSH concentration < 2.5^th^ percentile and an FT4 concentration within the normal range (as only two subjects had subclinical hyperthyroidism, these two groups were combined into a single hyperthyroidism group); and (4) isolated hypothyroxinemia was defined as an FT4 concentration < 2.5^th^ percentile and a TSH concentration within the normal range. Subjects with a TPO Ab concentration > 34 IU/mL were defined as TPO Ab-positive according to the measurement kit, and those with an TPO Ab concentration ≤ 34 IU/mL were defined as TPO Ab-negative.

### GDM

Each pregnant woman was subject to an OGTT test during her regular obstetric check-up during the 24^th^ to 28^th^ gestational weeks. Briefly, after an overnight fast, the blood glucose concentrations of subjects were determined at 0, 1, and 2 h after a 75-g dose of oral glucose. GDM was defined to exist if the subjects met any of the following criteria: a fasting blood glucose concentration of ≥ 5.1 mmol/L, a 1-h post-oral glucose blood-glucose concentration of ≥ 10.0 mmol/L, or a 2-h blood glucose post-oral glucose blood-glucose concentration of ≥ 8.5 mmol/L. The outcome and measurement time of GDM, as well as other potential covariates [age, body mass index (BMI), and parity] were independently reviewed by two researchers through examinations of the medical records and clinical results. The value of BMI at the first obstetric check-up was reviewed and used, because we were unable to obtained the pre-pregnant BMI information due to retrospective design of the study. In order to attenuate the influence of this situation, subjects with BMI obtained at the first obstetric check-up after 20 gestational weeks were excluded, and the gestational weeks of BMI measurement were further controlled.

### Statistical methods

Continuous variables are presented as means ± standard deviations (SD) or medians (interquartile) according to the normality. Student’s *t*-tests or non-parametric tests were used to detect differences in continuous variables between the TPO Ab-positive and TPO Ab-negative groups. Chi-square tests were used to compare the differences between the categorical variables of these two groups. The values of three indicators (the concentrations of FT4, TSH and TPO Ab) were divided into tertile groups, with the lowest group representing the lowest concentrations and the highest group representing the highest concentrations. Logistic regression analyses were performed to explore the associations between thyroid function and GDM. Multiple models were used for the adjustment: Model 1 was used for the univariate analysis, Model 2 was used to adjust for covariates (age, BMI, parity, and gestational weeks), and Model 3 was used to further adjust for the other two indicators of thyroid function. Logistic regression analyses were also performed to investigate the associations between different thyroid statuses (using euthyroidism as reference) or TPO Ab status (using TPO Ab negativity as a reference) and GDM. TPO Ab-positive subjects were excluded from the sensitivity analyses to exclude the potential influence of this status. All the analyses were performed using SPSS 20.0 (Chicago, IL, USA), and a two-sided *p* value < 0.05 was considered to indicate statistical significance. Power calculation were performed using data from our study with serum concentration of FT4 as the reference independent indicator and occurrence of GDM as the reference outcome indicators in total subjects. The prevalence of GDM in the reference group (bottom tertile) is 15.4%. Covariates were adjusted for in the analysis of logistic regression, contributing a R-squared value of 0.037. By setting a two-sided significance level of 0.05, with a final sample size of 2849 and a OR value of 0.71, the statistical power in our study is 86.0%. Calculations were operated using Pass 11.0 software (NCSS LLC, Utah, USA).

## Results

A total of 2849 pregnant women with a mean age of 30.0 ± 4.94 years were included in this retrospective study (Table 1). Among them, 381 (13.4%) developed GDM, and the other 2468 (86.6%) remained non-diabetic. A threshold TPO Ab concentration of > 34 IU/mL was defined as indicating TPO Ab-positivity; consequently, 235 (8.25%) subjects were observed to be TPO Ab-positive, and the remaining 2614 (91.75%) were TPO Ab-negative. TPO Ab-positive subjects tended to be older (mean: 30.8 vs. 29.9 years), have a higher BMI (median: 21.6 vs. 21.2 kg/cm^2^) and higher concentration of TSH (median: 1.66 vs. 1.41 mIU/L), and were more likely to be multiparous (parity ≥ 2: 47.7% vs. 37.1%) when compared with TPO-negative subjects. In contrast, no significant differences were observed in the gestational weeks of thyroid or BMI measurement, FT4 concentration, and proportion of GDM between TPO Ab-positive and TPO Ab-negative subjects (*p* = 0.289–1.000).

**Table 1:** Characteristic of subjects

	Total (*N* = 2849)	TPO Ab negativity (*N* = 2614)	TPO Ab positivity (*N* = 235)	*P*
Age, years	30.0 ± 4.94	29.9 ± 4.93	30.8 ± 4.97	**0.007**
BMI,kg/cm^2^	21.2 ± 3.01	21.2 ± 2.99	21.6 ± 3.11	**0.028**
Gestational weeks				
BMI	12.3 ± 2.40	12.3 ± 2.40	12.5 ± 2.41	0.289
FT4	15.9 ± 6.90	16.0 ± 6.90	15.9 ± 6.98	0.925
TSH & TPOAb	17.1 ± 3.59	17.1 ± 3.58	16.9 ± 3.74	0.514
Thyroid fuction				
FT4, pmol/L	14.3 (12.9, 15.8)	14.4 (13.0, 15.9)	14.1 (12.8, 15.8)	0.374
TSH, mIU/L	1.43 (0.93, 2.02)	1.41 (0.92, 1.98)	1.66 (0.99, 2.49)	** < 0.001**
TPOAb, IU/mL	12.8 (9.46, 18.5)	12.2 (9.22, 16.5)	96.4 (50.3, 204)	** < 0.001**
Parity				**0.002**
1	1767 (62.0)	1644 (62.9)	123 (52.3)	
≥ 2	1082 (38.0)	970 (37.1)	112 (47.7)	
Gestational diabetes mellitus, N (%)				1.000
Yes	381 (13.4)	350 (13.4)	31 (13.2)	
No	2468 (86.6)	2264 (86.6)	204 (86.8)	

*FT4* free tetraiodothyronine *TSH* thyroid-stimulating hormone *TPO Ab* thyroid peroxidase antibody;

As shown in Table 2, higher concentrations of FT4 (top vs. bottom tertile) was inversely associated with the risk of GDM. After adjustment for age, BMI, parity, and gestational weeks of thyroid function or BMI measurement, subjects with the highest FT4 concentrations (the *T3* group) had a 29% lower risk of GDM (vs. the *T1* group; *OR*: 0.71, *95% CI*: 0.54, 0.93). Similarly, these inverse associations persisted in the sensitivity analysis that excluded TPO Ab-positive subjects (Supplemental Table 1). No significant associations were observed between TSH concentrations and the GDM risk in any of the adjusted models and among either total subjects or TPO Ab-negative subjects. We further investigated the associations of extremely 2.5^th^/97.5^th^ or 10^th^/90^th^ percentile groups of FT4 and TSH with the risk of GDM, however, no significant results were found in total subjects (Table 3 & Supplemental Table 2) or subjects with TPO Ab-negativity (Supplemental Table 3 and 4).

**Table 2:** Associations between tertile of thyroid indicators and the risk of GDM

	T1	T2			T3		
	*Reference*	*OR*	*95%CI*	*P*	*OR*	*95%CI*	*P*
FT4, pmol/L ^a^	12.4 (11.6, 12.9)	14.4 (13.9, 14.8)			16.7 (15.9, 18.0)		
Model 1	1.00	0.83	0.64, 1.07	0.155	**0.72**	**0.55, 0.94**	**0.014**
Model 2	1.00	0.80	0.62, 1.04	0.801	**0.71**	**0.54, 0.93**	**0.014**
TSH, mIU/L ^a^	0.73 (0.47, 0.93)	1.43 (1.26, 1.61)			2.31 (2.03, 2.81)		
Model 1	1.00	1.02	0.79, 1.32	0.859	0.81	0.62, 1.06	0.123
Model 2	1.00	1.04	0.79, 1.36	0.779	0.87	0.65, 1.15	0.325

^a^presented as median (interquartile)

Logistic regression analysis were operated for exploration of associations. Model 1: without adjustment. Model 2: adjusted for age, BMI, parity, gestational week of thyroid indicators measurement, and gestational week of BMI

**Table 3:** Associations between extremely 2.5^th^/97.5^th^ percentile of thyroid indicators and the risk of GDM

	2.5%-97.5%	< 2.5%			> 97.5%			< 2.5%	> 97.5%		
	*Reference*	*OR*	*95%CI*	*P*	*OR*	*95%CI*	*P*	*Reference*	*OR*	*95%CI*	*P*
FT4, pmol/L											
Model 1	1.00	0.97	(0.69, 1.37)	0.864	0.88	(0.49, 1.59)	0.670	1.00	0.91	(0.47, 1.76)	0.770
Model 2	1.00	1.01	(0.71, 1.44)	0.964	0.86	(0.47, 1.58)	0.623	1.00	0.85	(0.43, 1.69)	0.646
TSH, mIU/L											
Model 1	1.00	1.04	(0.63, 1.74)	0.873	0.96	(0.33, 2.76)	0.941	1.00	0.92	(0.29, 2.95)	0.891
Model 2	1.00	0.92	(0.54, 1.56)	0.990	1.01	(0.34, 2.95)	0.990	1.00	1.10	(0.34, 3.59)	0.880

Logistic regression analysis were operated for exploration of associations. Model 1: without adjustment. Model 2: adjusted for age, BMI, parity, gestational week of thyroid indicators measurement, and gestational week of BMI

**Table 4:** Associations between thyroid status and the risk of GDM

	*Number of events/total (%)*	Model 1			Model 2		
		*OR*	*95%CI*	*P*	*OR*	*95%CI*	*P*
Thyroid function							
Euthyroid & TPO Ab- (reference group)	293/2208 (13.3)	1.00	-	-	1.00	-	-
Euthyroid & TPO Ab +	28/198 (14.1)	1.08	(0.71, 1.64)	0.730	0.98	(0.64, 1.51)	0.925
Subclinical hypothyroidism	3/27 (11.1)	0.82	(0.24, 2.73)	0.743	0.85	(0.25, 2.90)	0.794
Hyperthyroidism (including subclinical)	17/115 (14.8)	1.13	(0.69, 1.93)	0.642	1.00	(0.58, 1.72)	0.987
Isolated hypothyroxinaemia	40/301 (13.3)	1.00	(0.70, 1.43)	0.993	1.03	(0.72, 1.49)	0.858
TPO Ab positivity (vs. negativity)	235/2849 (8.2)	0.98	(0.66, 1.46)	0.932	0.88	(0.59, 1.32)	0.536
TSH ≥ 2.5 mU/L (vs. < 2.5 mIU/L)	369/2849 (13.0)	1.01	(0.73, 1.39)	0.967	1.12	(0.81, 1.56)	0.486

Logistic regression analysis were operated for exploration of associations. Model 1: without adjustment. Model 2: adjusted for age, BMI, parity, gestational week of thyroid indicators measurement, and gestational week of BMI

To further investigate the associations between thyroid function and GDM, subjects were divided into different thyroid-function groups (euthyroidism, subclinical hypothyroidism, hyperthyroidism, and isolated hypothyroxinemia) according to the percentile ranges of the FT4 and TSH concentrations. Compared with euthyroid subjects, no significant associations were found between the risk of GDM and subclinical hypothyroidism, hyperthyroidism, and isolated hypothyroxinemia (*p* = 0.536–0.987; Table 4). Similarly, no significant association was observed between TPO Ab-positivity (vs. TPO Ab-negativity) or higher TSH status (≥ 2.5 vs. < 2.5 mU/L) and the GDM risk (Table 4).

## Discussion

In this retrospective cohort study comprising 2849 Chinese pregnant women, higher FT4 concentrations tertile (instead of extremely 2.5^th^/97.5^th^ or 10^th^/90^th^ percentiles) was found to be associated with a lower risk of GDM. No significant associations were observed between TSH categories or status, thyroid function subtypes (vs. euthyroidism), TPO Ab-positivity (vs. -negativity), and the GDM risk. Results remained similar when subjects with TPO Ab-positivity were excluded in the sensitivity analyses.

We found that a higher concentration of FT4 (*T3* vs. *T1*) was independently associated with a lower risk of GDM. These results are consistent with those of a large retrospective study of 27,513 pregnant women in Shanghai, China, which found that 23% (*OR*: 0.77, *95% CI*: 0.68, 0.87) of subjects in the top FT4 concentration quintile had a lower GDM risk than those in the bottom quintile [20]. For subjects in the highest FT4 concentrations quintile, GDM occur less often in a prospective cohort study of 9209 subjects in the US [21]. In another case-controlled study conducted in the US, a higher concentration of free triiodothyronine (FT3), the biologically active thyroid hormone, or a higher FT3/FT4 ratio (top quartile vs. bottom quartile) was associated with a 3.89–13.6-fold increase in the GDM risk, whereas no significant independent association was found between the FT4 concentration and GDM risk [16]. The median FT4 concentrations of subjects were similar among these studies, ranging from 13.1 to 15.9 pmol/L [16, 20, 21], and our study subjects had the highest median FT4 concentration (16.7 pmol/L). These variations may be attributable to different sensitivities in the effects of thyroid hormones on GDM in Chinese and American women, given that the potential for racial heterogeneity was indicated in the former study [28]. The FT3/FT4 ratio tended to indicate the overt adverse risk for GDM better than FT3 concentration, as indicated in the former study [16]. Therefore, our discovery of the protective influence of a higher concentration of FT4 may be partly due to its reducing effect on the FT3/FT4 ratio. Unfortunately, the FT3 concentration data were not in the medical records, and the retrospective study design meant that this omission could not be remedied. Interestingly, in another cross-sectional study, lower concentrations of FT3, a lower FT3/FT4 ratio, and higher concentrations of FT4 were associated with a higher risk of T2DM [29]. Therefore, although GDM and T2DM are both characterized by impaired glucose homeostasis, thyroid hormones may affect these disorders differently. This aspect requires further exploration. Nevertheless, our study (together with former studies) might still indicated that achieving of a relative higher concentration of FT4 in the normal range during pregnancy should be encouraged for prevention of GDM.

The extremely 2.5^th^/97.5^th^ percentile of FT4 and TSH are also used to classify FT4 or TSH abnormality, we further explore their relationships with the risk of GDM. Since only a small part of subjects belong to the extremely 2.5^th^/97.5^th^ percentile groups, we further expand the classification to extremely 10^th^/90^th^ percentile of FT4 or TSH. Interestingly, unlike the FT4 concentration tertile, we did not observe significant associations between extremely 2.5^th^/97.5^th^ or 10^th^/90^th^ percentile of FT4 and the risk of GDM. One possible explanation might be that the numbers of subjects in tertile group are the same, which provided higher statistical efficiency than extremely 2.5^th^/97.5^th^ or 10^th^/90^th^ percentiles, and then more likely to detect significance results. Besides, it would be possible that the protective influence of higher FT4 might existed only among certain concentration range. Former studies seldom provided full-scale classification of FT4 and TSH as we did in our study. More studies might be needed to examine our results.

We observed a null association between the TSH concentration or status and GDM. This result was consistent with those of three previous studies (two in the US [16, 17], one in China [6]). However, three other studies reported adverse influences of higher TSH concentrations [13, 19, 22]. In a prospective study held in northern China including 7258 subjects and 594 GDM cases, TSH was positively associated with the GDM risk in subjects  with TSH concentrations ≤ 3.2 mIU/L, but not in those with TSH concentrations > 3.2 mIU/L[13]. Conversely, in a large retrospective study held in Shanghai, China, higher TSH concentrations (top quartile, > 1.96 mIU/L vs. bottom quartile, 0–0.52 mIU/L) were associated with a lower risk of GDM (*OR*: 0.78, *95% CI*: 0.69, 0.88) [20]. The differences in these studies’ designs and the cutoff points used for the classification of TSH concentrations increased the level of heterogeneity and the difficulty in comparing the results. Treatment with levothyroxine (L-T4) to decrease the concentration of TSH also led to inconsistent results [6, 12]. Thus, in a large prospective cohort study in Finland, treatment with L-T4 attenuated but did not eliminate the risk of GDM [12]. However, treatment with L-T4 doubled the risk of GDM in those with TSH concentrations of 2.5–4.8 mIU/L relative to the risk in those with TSH concentrations of 0.23–2.5 mIU/L [6]. These data indicate that the relationships between TSH concentrations and GDM are complex, and further studies are required to achieve a definitive understanding. Moreover, L-T4 treatments should be used cautiously, and other treatments must be identified.

The TPO Ab concentration is an important indicator of the existence of thyroid autoimmune disease. A certain TPO Ab concentration, which is defined in measurement kits, is used to define TPO Ab-positivity. Several studies have found that TPO Ab positivity (vs. negativity) is associated with a higher risk of GDM [14, 19, 26] and that this risk may be further amplified by the co-existence of SCH [24, 27]. In our study, 8.32% subjects were TPO Ab-positive, which was comparable to the proportions in previous studies [9, 30]. However, we did not observe a significant relationship between TPO Ab positivity (vs. negativity) and GDM, which was consistent with the findings of a prospective cohort study (1193 subjects) in the US [17] and a retrospective study (27,513 subjects) in Shanghai, China [20]. Our results, together with former studies, emphasized that conflict results existed between the association of TPO Ab positivity and GDM, and further studies were needed for better illustrate the problem.

Although previous studies have found that clinical hypothyroidism statuses (overt/sub-clinical/isolated) are associated with an increased GDM risk [12, 24, 25], we did not observe a significant relationship between the thyroid disorder status (hyperthyroidism or hypothyroidism vs. euthyroidism) and the GDM occurrence. This result is consistent with a prospective cohort study of 1683 subjects in Anhui, China [14]. Our results may be due to the fact that our study was performed in a city in southern China that is close to the sea, and iodized salt is commonly consumed due to the national salt iodization program. Therefore, the sufficiency of iodine in our study population might have prevented the adverse effect of hypothyroidism due to iodine deficiency [31]. These novel findings will enrich the literature, although more research is required to reach definitive conclusions.

This study had several strengths. First, serum thyroid indicators were measured before the diagnosis of GDM, and the retrospective cohort design enabled us to be certain of the temporal sequence of events and avoid a causal inversion. Second, by using multiple adjustment models, we avoided confounding effects and were able to identify the independent associations of each thyroid indicator with GDM. Third, sensitivity analyses were performed to remove the potential influence of TPO Ab-positivity, and the results of TPO Ab-positive subjects were excluded. Fourth, the use of the 2.5^th^ and/or 97.5^th^ percentiles of FT4 and TSH concentrations as thresholds for the definitions of clinical thyroid-disease subtypes enabled a better comparison or combination of our results with those of other researchers.

However, several limitations of our study merit careful consideration. First, the study was performed at a single obstetric center. Although this was the largest center in the city and covered a large population, future studies that include multiple centers are needed. Second, only three thyroid function indicators were measured, and we were unable to obtain information about the FT3 concentrations. Therefore, although a previous study showed that the influence of FT3 might be different to that of FT4 and demonstrated the potential clinical value of the FT3/FT4 ratio, we were unable to carry out a further exploration of this aspect. Third, we used retrospective data drawn from medical records, which did not contain the iodine concentrations in blood or urine. Therefore, we were unable to discern whether the associations between thyroid function and the GDM risk might have been mediated or influenced by the subjects’ nutritional iodine status. Further research is needed to examine these areas.

## Conclusions

In conclusions, this retrospective cohort study showed that higher FT4 concentrations tertile was associated with a lower risk of GDM in Chinese pregnant women. No significant associations were observed between the GDM status and the TSH concentrations, thyroid disorder status, and TPO Ab-positivity. Our results indicated that instead of simply focusing extremely percentiles or ill thyroid status, a relative lower FT4 concentration (even in the normal range) should also received more attention from the clinicians for the prevention of GDM. More well-designed prospective studies based on different populations are needed to examine and extend these findings.

## Supplementary Information

**Additional file 1: Table 1. **Associations between tertile of thyroid indicators and the risk of GDMin subjects with TPO Ab negativity. **Table 2.** Associations between extremely 10^th^/90^th^percentile of thyroid indicators and the risk of GDM. **Table 3.** Associations between extremely 2.5^th^/97.5^th^percentile thyroid indicators and the risk of GDM in subjects with TPO Abnegativity. **Table 4.** Associations between extremely 10^th^/90^th^percentile thyroid indicators and the risk of GDM in subjects with TPO Abnegativity.

## Data Availability

The data that support the findings of this study are available from the Affiliated Foshan Maternity & Child Healthcare Hospital, Southern Medical University, but restrictions apply to the availability of these data, which were used under license for the current study, and so are not publicly available. Data are however available from the authors upon reasonable request and with permission of Affiliated Foshan Maternity & Child Healthcare Hospital, Southern Medical University.
