# Supplementary material for: Associations between thyroid function and gestational diabetes mellitus in Chinese pregnant women: a retrospective cohort study
Source: BMC Endocr Disord. 2022 Feb 21;22:44. doi: 10.1186/s12902-022-00959-y (PMC8862524; doi:10.1186/s12902-022-00959-y)
Supplement: Supplementary file 1 — Additional file 1: Table 1. Associations between tertile of thyroid indicators and the risk of GDMin subjects with TPO Ab negativity. Table 2. Associations between extremely 10th/90thpercentile of thyroid indicators and the risk of GDM. Table 3. Associations between extremely 2.5th/97.5thpercentile thyroid indicators and the risk of GDM in subjects with TPO Abnegativity. Table 4. Associations between extremely 10th/90thpercentile thyroid indicators and the risk of GDM in subjects with TPO Abnegativity. [file 12902_2022_959_MOESM1_ESM.docx]

**Supplemental Table 1**. Associations between tertile of thyroid indicators and the risk of GDM in subjects with TPO Ab negativity.

|  | T1 |  | T2 | | |  | T3 | | |
| --- | --- | --- | --- | --- | --- | --- | --- | --- | --- |
|  | *Reference* |  | *OR* | *95%CI* | *P* |  | *OR* | *95%CI* | *P* |
| FT4, pmol/L ^a^ | 12.4 (11.6, 12.9) |  | 14.3 (13.9, 14.8) | | |  | 16.7 (15.9, 18.0) | | |
| Model 1 | 1.00 |  | 0.84 | 0.64, 1.10 | 0.200 |  | **0.72** | **0.55, 0.95** | **0.021** |
| Model 2 | 1.00 |  | 0.81 | 0.62, 1.07 | 0.143 |  | **0.71** | **0.53, 0.94** | **0.018** |
| TSH, mIU/L ^a^ | 0.73 (0.48, 0.93) |  | 1.43 (1.26, 1.61) | | |  | 2.28 (2.02, 2.74) | | |
| Model 1 | 1.00 |  | 1.03 | 0.79, 1.34 | 0.839 |  | 0.82 | 0.62, 1.08 | 0.162 |
| Model 2 | 1.00 |  | 1.04 | 0.78, 1.37 | 0.811 |  | 0.88 | 0.66, 1.19 | 0.408 |

^a^: presented as median (interquartile).

Logistic regression analyses were operated for exploration of associations. Model 1: without adjustment. Model 2: adjusted for age, BMI, parity, gestational week of thyroid indicators measurement, and gestational week of BMI.

**Supplemental Table 2**. Associations between extremely 10^th^/90^th^ percentile of thyroid indicators and the risk of GDM.

|  | 10%-90% |  | <10% | | |  | >90% | | |  | <10% |  | <10% | | |
| --- | --- | --- | --- | --- | --- | --- | --- | --- | --- | --- | --- | --- | --- | --- | --- |
|  | *Reference* |  | *OR* | *95%CI* | *P* |  | *OR* | *95%CI* | *P* |  | *Reference* |  | *OR* | *95%CI* | *P* |
| FT4, pmol/L |  |  |  | | |  |  | | |  |  |  |  | | |
| Model 1 | 1.00 |  | 0.93 | (0.64, 1.34) | 0.695 |  | 0.83 | (0.57, 1.22) | 0.342 |  | 1.00 |  | 1.08 | (0.74, 1.56) | 0.695 |
| Model 2 | 1.00 |  | 0.88 | (0.60, 1.29) | 0.507 |  | 0.85 | (0.57, 1.26) | 0.409 |  | 1.00 |  | 0.96 | (0.57, 1.62) | 0.886 |
| TSH, mIU/L |  |  |  | | |  |  | | |  |  |  |  | | |
| Model 1 | 1.00 |  | 0.95 | (0.66, 1.37) | 0.777 |  | 0.93 | (0.64, 1.34) | 0.695 |  | 1.00 |  | 1.06 | (0.73, 1.53) | 0.777 |
| Model 2 | 1.00 |  | 0.94 | (0.64, 1.39) | 0.769 |  | 1.05 | (0.72, 1.53) | 0.817 |  | 1.00 |  | 1.11 | (0.72, 1.56) | 0.769 |

Logistic regression analyses were operated for exploration of associations. Model 1: without adjustment. Model 2: adjusted for age, BMI, parity, gestational week of thyroid indicators measurement, gestational week of BMI.

**Supplemental Table 3**. Associations between extremely 2.5^th^/97.5^th^ percentile thyroid indicators and the risk of GDM in subjects with TPO Ab negativity.

|  | 2.5%-97.5% |  | <2.5% | | |  | >97.5% | | |  | <2.5% |  | >97.5% | | |
| --- | --- | --- | --- | --- | --- | --- | --- | --- | --- | --- | --- | --- | --- | --- | --- |
|  | *Reference* |  | *OR* | *95%CI* | *P* |  | *OR* | *95%CI* | *P* |  | *Reference* |  | *OR* | *95%CI* | *P* |
| FT4, pmol/L |  |  |  | | |  |  | | |  |  |  |  | | |
| Model 1 | 1.00 |  | 0.99 | (0.69, 1.41) | 0.947 |  | 0.90 | (0.49, 1.66) | 0.732 |  | 1.00 |  | 0.91 | (0.46, 1.82) | 0.786 |
| Model 2 | 1.00 |  | 1.01 | (0.70, 1.46) | 0.948 |  | 0.84 | (0.44, 1.58) | 0.583 |  | 1.00 |  | 0.83 | (0.41, 1.69) | 0.601 |
| TSH, mIU/L |  |  |  | | |  |  | | |  |  |  |  | | |
| Model 1 | 1.00 |  | 1.16 | (0.69, 1.94) | 0.578 |  | 0.65 | (0.15, 2.79) | 0.562 |  | 1.00 |  | 0.56 | (0.12, 2.61) | 0.461 |
| Model 2 | 1.00 |  | 1.03 | (0.60, 1.76) | 0.913 |  | 0.77 | (0.18, 3.37) | 0.728 |  | 1.00 |  | 0.75 | (0.16, 3.56) | 0.715 |

Logistic regression analyses were operated for exploration of associations. Model 1: without adjustment. Model 2: adjusted for age, BMI, parity, gestational week of thyroid indicators measurement, and gestational week of BMI.

**Supplemental Table 4**. Associations between extremely 10^th^/90^th^ percentile thyroid indicators and the risk of GDM in subjects with TPO Ab negativity.

|  | 10%-90% |  | <10% | | |  | >90% | | |  | <10% |  | <10% | | |
| --- | --- | --- | --- | --- | --- | --- | --- | --- | --- | --- | --- | --- | --- | --- | --- |
|  | *Reference* |  | *OR* | *95%CI* | *P* |  | *OR* | *95%CI* | *P* |  | *Reference* |  | *OR* | *95%CI* | *P* |
| FT4, pmol/L |  |  |  | | |  |  | | |  |  |  |  | | |
| Model 1 | 1.00 |  | 0.95 | (0.65, 1.39) | 0.791 |  | 0.86 | (0.58, 1.27) | 0.855 |  | 1.00 |  | 0.90 | (0.54, 1.51) | 0.691 |
| Model 2 | 1.00 |  | 0.90 | (0.61, 1.33) | 0.590 |  | 0.85 | (0.57, 1.28) | 0.849 |  | 1.00 |  | 0.95 | (0.55, 1.62) | 0.838 |
| TSH, mIU/L |  |  |  | | |  |  | | |  |  |  |  | | |
| Model 1 | 1.00 |  | 0.95 | (0.64, 1.39) | 0.781 |  | 0.94 | (0.63, 1.40) | 0.748 |  | 1.00 |  | 0.99 | (0.58, 1.68) | 0.967 |
| Model 2 | 1.00 |  | 0.95 | (0.64, 1.43) | 0.813 |  | 1.08 | (0.71, 1.63) | 0.728 |  | 1.00 |  | 1.13 | (0.65, 1.97) | 0.665 |

Logistic regression analyses were operated for exploration of associations. Model 1: without adjustment. Model 2: adjusted for age, BMI, parity, gestational week of thyroid indicators measurement, and gestational week of BMI.

.
